# Ergocalciferol improves endothelial vasodilatory and vasoconstrictor function in an *in vivo* model of mild uraemia

**DOI:** 10.1042/BSR20190711

**Published:** 2019-12-17

**Authors:** Gavin Dreyer, Julius Kieswich, Steven Harwood, Amrita Ahluwalia, Muhammad M. Yaqoob

**Affiliations:** 1 William Harvey Research Institute of Queen Mary University, John Vane Science Building, Charterhouse Square Campus, London EC1B 6BQ, U.K.; 2 Department of Vascular Pharmacology, William Harvey Research Institute of Queen Mary University, John Vane Science Building, Charterhouse Square Campus, London EC1B 6BQ, U.K.; 3 Diabetic Kidney Centre and Renal Unit, Barts Health NHS Trust, Royal London Hospital, Whitechapel Road, London E1 1BB, U.K.

**Keywords:** chronic kidney disease, endothelial function, ergocalciferol

## Abstract

Endothelial dysfunction and vitamin D deficiency are prevalent in patients with cardiovascular disease (CVD) and chronic kidney disease (CKD). Both are risk factors for cardiovascular events in patients with CKD. No studies have investigated the effect of nutritional forms of vitamin D on endothelial function in earlier stages of CKD, when vascular endothelium may be more amenable to this therapy. We studied the effect of ergocalciferol in a pre-clinical model of mild uraemia.

Male Wistar rats underwent either a 5/6th nephrectomy or sham surgery. Four weeks after the final stage of the surgery, these two groups were randomly allocated to placebo or an oral dose of 1000 iu of ergocalcfierol at day 7 and 2 pre sacrifice. Vascular responses to acetylcholine, Spermine NONOate and phenylephrine were determined in aortic rings. Blood pressure, calcium, phosphate and parathyroid hormone were measured in all groups.

Ergocalciferol significantly improved the endothelium-dependent responses to acetylcholine and overcame the blunting of the contractile response to phenylephrine seen in uraemic animals. Ergocalciferol improved the contractile response to potassium chloride in uraemic, but not sham animals. All effects occurred independently of changes to calcium, phosphate, parathyroid hormone and systolic blood pressure. There were no differences in endothelium-independent relaxation to Spermine NONOate.

In summary, in a model of mild uraemia, ergocalciferol improved vasodilator and vasoconstrictor tone independently of blood pressure and bone mineral parameters suggesting a direct effect of ergocalciferol on the endothelium.

## Introduction

Chronic kidney disease (CKD) and concomitant vitamin D deficiency (VDD) is a common clinical entity that may result in an increased risk of cardiovascular disease (CVD) [1–7]. CVD remains the most common cause of death in patients with all stages of CKD, including patients after renal transplantation. Chronic kidney disease, even in its earlier stages, is now recognized as a strong cardiovascular (CV) risk factor, independent of diabetes mellitus and hypertension [8]. Progression to end-stage kidney disease (ESKD) in patients with CKD is far less common than death from CVD, the risk of which is increased even in mild CKD [9,10] and accounts for approximately 40% of all deaths in patients with ESKD [11,12].

Endothelial dysfunction is the consequence of a complex interplay of traditional and non-traditional risk factors and is strongly associated with an elevated risk of CVD in CKD alone and when CKD and VDD coexist [13–16]. We and others have demonstrated that therapeutic intervention with vitamin D has the potential to ameliorate endothelial dysfunction when CKD and VDD co-exist [17–19].

The majority of studies of vitamin D status or therapy in CKD have included patients treated with haemodialysis or used vitamin D receptor agonists (VDRA) [1–5]. Investigating the effect of vitamin D on endothelial dysfunction in the earlier stages of CKD is logical since the function of the vascular endothelium is already abnormal and is likely to be more responsive to therapeutic intervention compared with ESKD. Therapeutic intervention with nutritional vitamin D compounds such as ergocalciferol rather than VDRA in early CKD and concomitant VDD is recommended by international guidelines [20,21]. Ergocalciferol is cheap (compared with the expense of newer active vitamin D receptor activators), safe, and well tolerated and can be effectively used to increase serum 25 (OH) D concentrations in CKD and suppress parathyroid hormone (PTH) [22,23].

We and others have previously demonstrated that ergocalciferol increases endothelium-dependent vasodilatation in the setting of a randomized controlled trial [17,24] and that the putative mechanism for this includes increased eNOS expression and nitrite production in cultured human aortic endothelial cells (HAEC) [17]. However, the *in vitro* experiments were not conducted in a uraemic milieu and did not address the effect of ergocalciferol on functional changes to the vascular endothelium in a pre-clinical model of mild uraemia.

*In vivo* studies that have evaluated the effect of vitamin D compounds on endothelial function in experimental uraemia have principally used models of advanced uraemia rather than models of the earlier stages of CKD and have used VDRA rather than nutritional vitamin D compounds [25–28]. Given that vitamin D compounds have a range of pleiotropic effects [29], there is no pecuniary reason for each vitamin D compound to act on similar cellular pathways or to generate the same magnitude of effect. This concept is supported by the work of Dong et al. [30], Borges et al. [31,32] and Wong et al*.* [33] which has alluded to potential differential mechanistic effects of both nutritional and activated vitamin D compounds on endothelial cellular responses.

The effect of ergocalciferol on endothelial function in experimental models of uraemia, and particularly mild uraemia reflecting the earlier stages of CKD, remains unclear. Supporting our previous clinical and *in vitro* studies demonstrating the beneficial effect of ergocalciferol on endothelial function, we describe how ergocalciferol affects vasoconstrictor function and endothelium-dependent vasodilator responses in a pre-clinical model of mild CKD.

## Methods

All animal experiments were conducted according to the Animals (Scientific Procedures) Act 1986, U.K. The animal protocols followed in the present study were approved by the Animal Welfare Ethics Review Board (AWERB) of Queen Mary University of London in accordance with the derivatives of both the Home Office guidance on the Operation of Animals (Scientific Procedures Act 1986) published by Her Majesty’s Stationery Office and the Guide for the Care and Use of Laboratory Animals of the National Research Council. The present study was performed under licence issued by the Home Office (Procedure Project Licence; 70/7055). Reagents were purchased from Sigma Aldrich U.K. unless stated otherwise. Male Wistar rats (approximately 200–250 g, Charles River, U.K.) were fed on a standard rodent diet containing 18.7% protein, 1.0% Ca^2+^, 0.7% NaCl and 3.6 IU cholecalciferol/g (Lillico Biotechnology, U.K.) with free access to water. Animals were subjected to a 5/6th nephrectomy (SNx) for the induction of uraemia in a two stage procedure. This model has been shown to result in uraemia with an associated endothelial dysfunction [34].

### Anaesthesia and surgical technique

In the first stage of surgery, animals were anaesthetised by an intra-peritoneal injection consisting of 100 mg/ml of ketamine hydrochloride, and 23.3 mg/ml of xylazine hydrochloride made up in a 2:1 ratio (ketamine:xylazine) at a dose of 1.5 ml/kg. The left flank of the animal was shaved and an incision made The left kidney was exposed, isolated and de-capsulated using small blunt forceps and two-thirds of the kidney mass was removed. Upon cessation of bleeding, the renal remnant was replaced in its original anatomical position. Isotonic saline (2 ml) was introduced into the peritoneum to replace fluid lost during surgery. Analgesia was provided pre and post-operatively with Vertegesic, 0.03 ml (buprenorphine, 0.342 mg/ml, (Alstoe Animal Health, York, U.K.)). Ten days later, SNx animals underwent the second stage of the renal mass reduction procedure. Animals were prepared in the same way and the right kidney was completely removed. Control animals underwent the same procedure without removal of the renal mass however at both stages the renal capsule was removed.

### Therapeutic intervention with ergocalciferol

Animals received either ergocalciferol 1000 IU (UCB Pharma, U.K.), or an equivalent volume of vehicle (ethyl oleate) by oral gavage at 7 and 2 days before killing in line with bolus dosing of ergocalciferol in guidelines [20] and to reduce stress on the animals. We designed a randomization schedule that generated four experimental groups – sham animals (*n* = 12), of whom half received ergocalciferol and half vehicle and SNx animals (*n* = 12) of whom half received ergocalciferol and half vehicle. The research team (GD, MMY) were blinded to the randomisation schedule.

### Measurement of blood pressure

Arterial blood pressure was measured in separate experiments involving the same four experimental groups, study compound dosing and duration of uraemia. After anaesthesia, a tracheostomy was performed and an arterial catheter to monitor pulse and blood pressure was inserted into the right carotid artery. Blood pressure was measured by attaching the catheter to a pressure transducer (Harvard Apparatus, Kent, U.K.). Blood pressure was recorded for at least 20 min until a stable value was obtained.

### Killing of animals and preparation of aortic rings

Four weeks following the final stage of the SNx surgery, the animals not used for blood pressure measurement were killed. The thoracic and abdominal aorta was removed and immediately immersed in a bath of standard Krebs-Ringer solution containing NaCl 69 g/l, KCl 3.5 g/l, MgSO_4_^.^7H_2_0 2.9 g/l, KH_2_PO_4_ 1.6 g/l, NaHCO_3_, 21 g/l, D-glucose (anhydrous) 20 g/l and 2.5 ml/l of 1 mmol/l CaCl_2_. The aorta was cleaned of all non-vascular tissue and individual ring segments of approximately 7 mm were prepared. Aortic rings were suspended in Krebs-Ringer solution as previously described in a 10 ml organ bath apparatus that was heated to 37°C The aortic rings were suspended on appropriate wires and connected to Lab Chart Reader (ADInstruments, Oxford, U.K.) by an ADInstruments octabridge at 1 g of resting tension. The Krebs-Ringer solution was aerated with Carbogen (5% CO_2_, 95% O_2_) throughout.

### Experimental procedures performed on isolated rat aortic rings

**Figure 1:**
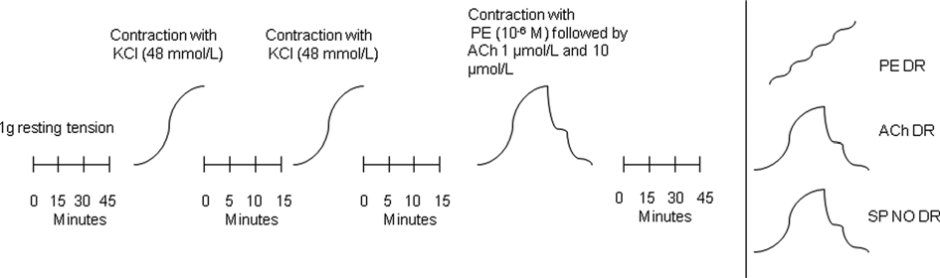
Tissue preparation (before long vertical line) and experimental procedures (after long vertical line) Short vertical hatch lines represent double rinse of aortic ring with Krebs-Ringer solution each spaced 5 min apart. Experimental procedures were conducted on individual aortic rings after each ring had undergone tissue preparation procedures. Each ring was subjected to one experimental compound only. The number of aortic rings for each experiment is described in Table 2. DR = dose response. PE = phenylephrine, ACh = acetylcholine, SpNO = spermine NONOate.

Tissue preparation and assessment is shown in Figure 1. Following equilibration for 45 min at a resting tension of 1 g aortic rings were pre-contracted twice with 48 mmol/l KCl with 3 × 15-min wash intervals in between. Following this aortic rings were pre-contracted with 10 μM phenylephrine (PE) and then sequential concentrations of acetylcholine (ACh) (1 and 10 µmol/l) applied to assess endothelium integrity. Endothelial function was considered intact if aortic rings demonstrated > 50% relaxation. Prior to the addition of experimental compounds, aortic rings were pre-contracted with PE to achieve a contractile tension of 90% of the response to the second dose of 48 mmol/l KCl. Cumulative concentration response curves were generated for Ach (0.001–30 µmol/l), spermine NONOate (SpNO, SpNO 0.001–30 µmol/l) (Cayman Chemical, U.K.) or PE (0.001–30 µmol/l).

### Measurement of biochemical parameters

Blood was collected on day 0 (killing) and assayed for urea, creatinine, Ca^2+^ and PO_4_ using an automated analyser (IDEXX Laboratories Ltd, Horsham, West Sussex, U.K.). Serum PTH concentrations were assayed using an ELISA kit (ALPCO Diagnostics, Stratech Scientific Ltd, Newmarket, Suffolk, U.K.). Serum 25 (OH) D concentrations were assessed by UPLC-MS at the Royal London Hospital.

### Statistical analysis

Aortic ring tension is expressed in grams (g). The response of aortic rings to KCl to assess reproducibility of contraction is expressed as change in tension in g above resting baseline. Relaxation of aortic rings induced by ACh or SpNO is expressed as the percentage relaxation after pre-determined contractile tension was achieved. The two-way ANOVA test with repeated measures was used to assess differences between concentration response curves [35]. Continuous data were compared using the Student’s *t*-test or Mann–Whitney *U* test depending on the distribution of the data. A one-way ANOVA test was used for comparison between multiple groups. The effect of agonist response is expressed as the −log EC_50_ (pEC_50_) and compared with a Student’s *t* test. Data are presented as mean ± standard error (SEM). The “*n*” refers to the number of rats per experiment. Statistical analysis was performed using GraphPad Prism software (version 5, U.S.A.). A *P* value of < 0.05 was considered to represent statistical significance.

## Results

### The effect of ergocalciferol on baseline rat physiology and biochemistry

One animal was lost prior to killing in the SNx group. The SNx model effectively induced uraemia as evidenced by the higher serum creatinine in SNx compared with sham operated animals (sham serum creatinine 40.1 ± 1.0 µmol/l versus SNx serum creatinine 95.1 ± 3.5 µmol/l, *P* < 0.0001, *n* = 5)). Serum creatinine did not differ between vehicle and ergocalciferol treated animals in sham (38.8 ± 3.2 µmol/l versus 41.4 ± 2.5 µmol/l, *P* = 0.18) or uraemic experimental groups (95.1 ± 12.9 µmol/l versus 98.5 ± 13.6 µmol/l, *P* = 0.65). Therapeutic intervention with ergocalciferol compared to vehicle significantly increased serum 25 (OH) D concentrations in both sham (44.4 ± 7.5 nmol/l versus 92.6 ± 16.8 nmol/l, *P* = 0.0004, *n* = 5) and SNx animals (53.0 ± 8.2 nmol/l versus 82.1 ± 17.4 nmol/l, *P* = 0.005, *n* = 5) with no significant difference between groups at baseline (*P* = 0.12).

Serum calcium (*P* = 0.09), phosphate (*P* = 0.64) and PTH (*P* = 0.63) did not differ significantly between experimental groups (Table 1). There was no significant difference between experimental groups in systolic blood pressure (*P* = 0.39, Table 1). Two days prior to killing, animal weights differed between sham and uraemic rats (sham weight 444 g ± 12 versus SNx weight 406 g ± 6, *P* = 0.006, *n* = 6) but there were no differences between vehicle and ergocalciferol treated animals in sham (vehicle weight 427 ± 8 versus ergocalciferol weight 461 g ± 21, *P* = 0.17, *n* = 6) and uraemic (vehicle weight 398 g ± 6 versus ergocalciferol weight 414 g ± 10, *P* = 0.19, *n* = 6) groups.

**Table 1:** Serum calcium, phosphate and parathyroid hormone (PTH) and blood pressure (BP) at killing

	Sham vehicle	Sham ergocalciferol	SNx vehicle	SNx ergocalciferol	*P* value
**Serum calcium (mmol/l)**	2.54 ± 0.01	2.57 ± 0.07	2.87 ± 0.4	2.73 ± 0.14	0.09
**Serum phosphate (mmol/l)**	2.89 ± 0.30	3.03 ± 0.62	3.54 ± 1.60	3.49 ± 0.95	0.64
**Serum PTH (pg/ml)**	37.8 ± 8.2	45.6 ± 12.8	35.5 ± 7.4	40.3 ± 18.9	0.63
**Systolic BP (mm Hg)**	133 ± 25	135 ± 29	123 ± 16	115 ± 21	0.39

Mean ± SEM *P* value calculated using one way ANOVA test; *n* = 5 per group

### Endothelium-dependent relaxation responses

**Figure 2:**
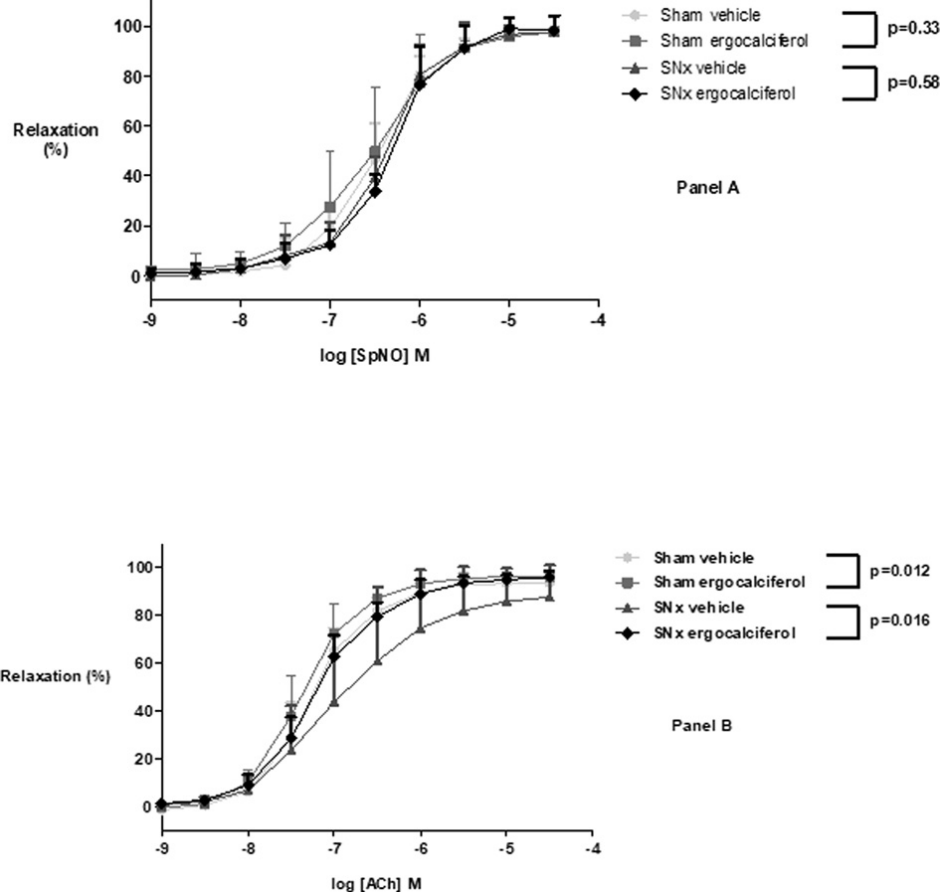
Tissue exposure to Spermine NONO and Acetylcholine Panel (**A**) exposure to Spermine NONO - Tissue relaxation did not significantly differ between vehicle or ergocalciferol treated animals in either the sham (two-way ANOVA with repeated measures, *P* = 0.33) or SNx groups (two-way ANOVA with repeated measures, *P* = 0.58); *n* = 5 per group. Panel (**B**) exposure to acetylcholine - Ergocalciferol compared to vehicle improved endothelial relaxation in both sham (two-way ANOVA with repeated measures, *P* = 0.012) and SNx animals (two-way ANOVA with repeated measures, *P* = 0.016); *n* = 5 per group

There was a significant overall beneficial effect of ergocalciferol compared with vehicle on endothelial function as evidenced by increased aortic ring vasodilatation after the addition of ACh following pre-contraction with PE in both sham (*P* = 0.012) and SNx animals (*P* = 0.016) (Figure 2). A summary of the pEC_50_ and maximum achieved aortic ring relaxation is shown in Table 2.

**Table 2:** The effect of ergocalciferol on the response to vasodilators and vasoconstrictors in sham and SNx operated animals

		Sham			SNx		
Treatment		ACh, *n* = 5	SpNO, *n* = 5	PE, *n* = 5	ACh, *n* = 5	SpNO, *n* = 5	PE, *n* = 5
**Vehicle**	**pEC_50_**	7.3 ± 0.06	6.5 ± 0.06	6.8 ± 0.06	6.9 ± 0.04	6.4 ± 0.1	6.8 ± 0.07
	**Max**	97.8 ± 2.7%	96.5 ± 2.6%	4.0 ± 0.07 g	87.8 ± 1.4%	98.7 ± 5.2%	3.5 ± 0.08 g
**Ergocalciferol**	**pEC_50_**	7.4 ±0.07	6.5 ± 0.06	6.7 ± 0.07	7.2 ± 0.04	6.2 ± 0.1	6.4 ±0.07
	**Max**	97.9 ± 2.0%	98.3 ± 2.7%	4.3 ± 0.09 g	95.8 ± 1.3%	96.6 ± 5.4%	4.2 ± 0.08 g*

* significant at *P* = 0.03 compared with corresponding vehicle in the SNx group

ACh - acetylcholine, SpNO = spermine NONOate, PE - phenylephrine, Max - either maximum relaxation as a percentage of relaxation after preconstruction with PE or maximum achieved tension in g. Data are mean ± SEM. pEC_50_ - negative logarithm of the EC_50_. n numbers reflect the number of individual aortic ring segments per experiment.

The addition of SpNO generated almost complete relaxation of aortic tissue after pre-contraction with PE. The response to the NO donor did not differ significantly between vehicle or ergocalciferol treated animals in either the sham (*P* = 0.33) or SNx groups (*P* = 0.58) (Figure 2).

### Vasoconstrictor responses

**Figure 3:**
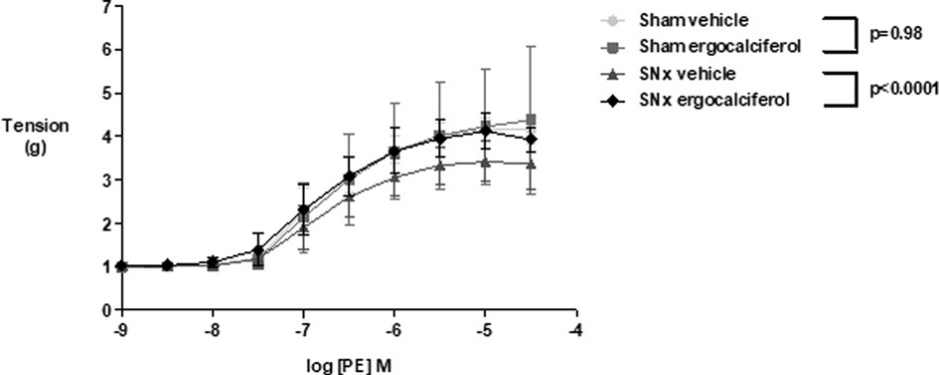
Contractile resposne to phenylephrine between treatment groups In the SNx group, ergocalciferol significantly increased overall contractile response to PE compared with vehicle (two-way ANOVA with repeated measures *P* < 0.0001) but there was no difference in contractile response to PE between vehicle and ergocalciferol in the sham group (two-way ANOVA with repeated measures *P* = 0.98); *n* = 5 per group.

There was no difference in contractile response to PE between vehicle and ergocalciferol treated animals in the sham surgery group (*P* = 0.98). In the SNx group, ergocalciferol significantly increased overall contractile response to PE compared with vehicle (*P* < 0.0001) (Figure 3). The maximum achieved tension (g) was increased in the SNx group animals treated with ergocalciferol compared to SNx animals treated with vehicle whilst the potency remained unchanged (*P* = 0.03, Table 2).

**Figure 4:**
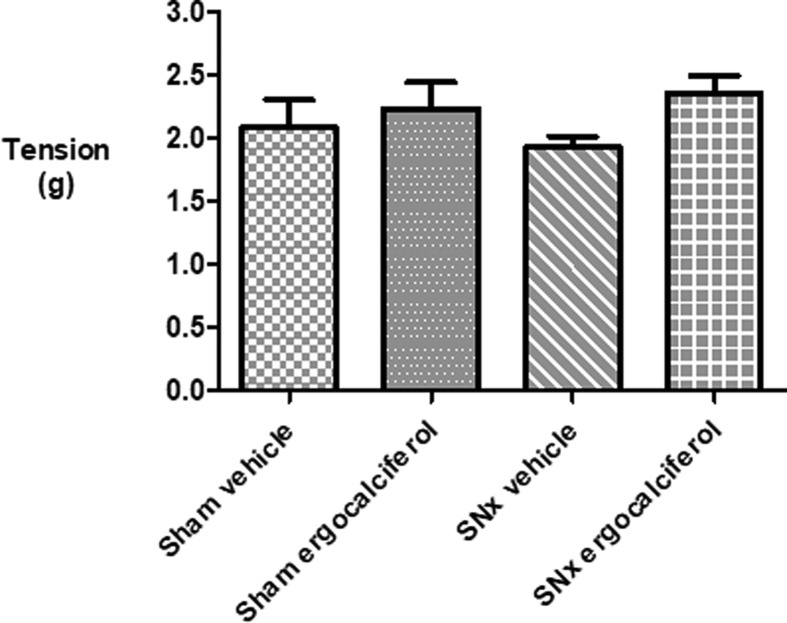
Contractile response to the addition of 48 mmol KCl to organ baths Sham animals vehicle 2.1 ± 0.2 g versus ergocalciferol 2.2 ± 0.2 g, *P* = 0.63. SNx animals vehicle 1.9 ± 0.1 g versus ergocalciferol 2.4 ± 0.1 g, *P* = 0.02.

There was an equivalent magnitude of contraction above baseline resting tension after the addition of 48 mmol/l KCl in sham animals treated with either vehicle or ergocalciferol. In uraemic animals, the contraction response to KCl was statistically greater in ergocalciferol treated animals compared with vehicle treated animals (Figure 4).

## Discussion

In an *in vivo* model of mild uraemia, we describe how ergocalciferol compared to vehicle improved the endothelial vasodilator response to ACh. In addition, ergocalciferol improved endothelial vasodilatory function in sham operated animals. This finding implies that increasing vitamin D levels even in a non-uraemic milieu, which were similar to uraemic animals, can enhance endothelial function and overcome the detrimental effect of uraemia on the vascular tree. There were no significant differences in the vasodilator response in either sham or SNx animals in response to the direct NO donor, SpNO, indicating that ergocalciferol improved vasodilator function through an effect on the endothelium and not through alteration of smooth muscle relaxant pathways. Uraemia blunted the contractile response to the adrenoceptor agonist PE but also to the depolarizing stimulus of KCl, and both of these were reversed in SNx animals treated with ergocalciferol, returning contractile function to the level seen in sham operated rats. Since the relaxant response to SpNO was unchanged the findings with the contractile agents suggest that ergocalciferol may be acting to alter contractile tone through effects upon smooth muscle depolarizing pathways.

Systolic blood pressure in sham operated and SNx rats was not significantly different. Similarly, systolic blood pressure did not differ in these experimental groups between animals treated with either vehicle or ergocalciferol. In SNx animals treated with vehicle compared with ergocalciferol, both endothelial vasorelaxant and vasoconstrictor responses were reduced compared with animals treated with ergocalciferol. The effect of the reduction of vasoconstrictor tone may represent a compensatory mechanism in the early stages of uraemia that prevents the development of systolic hypertension and may be mediated by a reduction in availability of noradrenaline in uraemia [36]. In addition, a reduction in both vasoconstrictor and vasodilator tone may represent impaired auto-regulatory function of the arterial tree which could expose end organ capillary beds to variations in systemic systolic blood pressure and consequent tissue damage.

We show that in SNx rats, ergocalciferol not only improved the endothelium-dependent relaxation responses but also normalized the vascular contractile response to two distinct stimuli. The observation that SNx rats treated with ergocalciferol have contractile responses to PE almost normalized to those in sham operated animals implies that uraemia desensitizes the target of PE (α-adrenergic receptor) and that ergocalciferol overcomes this desensitization. Our findings suggest that ergocalciferol has a beneficial effect on vascular function both through improving the endothelial vasodilator response and by restoring normal contractile function and therefore vascular auto-regulation. The exact mechanism by which this process occurs is unclear but may include restoration of noradrenaline release by ergocalciferol, an increase in alpha adrenergic receptor expression via a genomic effect of ergocalciferol or an improvement in the function of existing adrenergic receptors through a post translational modification by ergocalciferol. The mechanism by which ergocalciferol increases the contractile response to KCl requires further study but possible explanations include changes in smooth muscle bulk in vessel walls [37] or changes calcium channel function.

It would be expected that untreated uraemic animals would go on to develop systolic hypertension as a consequence of progressive kidney failure. The fact that systolic blood pressure was not different between sham operated and SNx rats and that ergocalciferol did not reduce systolic blood pressure in SNx rats may reflect the relatively short duration of uraemia and the relatively short duration of therapy with ergocalciferol in these studies. Equally, this may reflect the concomitant improvement in both vasodilator and vasoconstrictor tone which both maintains systemic blood pressure and, by restoring vascular homeostasis, protects end organs from tissue damage from fluctuations in systemic blood pressure. Myogenic tone in uraemic rat middle cerebral arteries has been shown to be impaired, exposing the brain to variations in systemic blood pressure [38]. Thus restoring vascular homeostasis in uraemia has the potential to protect critical organ beds.

The findings reported here support those of our previous experimental work [17]. Our randomised controlled trial demonstrated that the iontophoresis of ACh but not sodium nitroprusside improved the function of the microcirculatory endothelium of the skin in non-diabetic, normotensive patients with CKD stage 3-4 and concomitant VDD when treated with ergocalciferol compared to placebo. In addition, we demonstrated that ergocalciferol upregulated eNOS gene expression, increased the expression of eNOS protein and increased the concentration of nitrite in the supernatant of cultured HAEC. The observation that ergocalciferol improves endothelium-dependent relaxation after the addition of ACh in our *in vivo* model of mild uraemia suggests that ergocalciferol is potentially exerting an effect on the endothelium through a similar mechanism involving eNOS expression and function.

In the current study and our previous clinical and experimental studies [17], the improvements in endothelial function after treatment with ergocalciferol occurred independently of changes in blood pressure, Ca^2+^, PO_4_ and PTH. Jolma et al*.* [39,40] and Koobi et al. [41] have demonstrated that endothelium-dependent relaxant responses are improved by increasing dietary supplementation of calcium in experimental uraemia through effects on the calcium-dependent K^+^ channel. Serum calcium did not change in the current study or our previous study [17] and this supports the concept that ergocalciferol is exerting a direct and beneficial effect on endothelial function independently of serum calcium, PTH and phosphate concentrations. The effect of ergocalciferol on vascular function in uraemia is therefore potentially multi-factorial involving not only increased expression and function of eNOS but also an effect on the calcium-dependent potassium channel and α-adrenergic receptors. This effect may be mediated through transcriptional or post-translation modification, but further studies are required to confirm this.

Additional *in vivo* models of differing degrees of uraemia can be achieved through the use of an adenine rich diet [43] or a varied surgical approach as described by Baracho et al. [44]. At the time of designing our experiments, there were no studies which examined a shorter duration of uraemia, reflecting earlier stage kidney disease, where the response of the vasculature will be different to that of more advanced kidney disease. We have addressed this by killing animals at 4 weeks following completion of nephrectomy surgery compared with other studies where the total duration of uraemia has ranged from 8 to 24 weeks [27,39,40,42].

## Summary

Given the strong association of endothelial dysfunction with CVD in patients with CKD [13,14,45], the results of these descriptive *in vitro* and clinical experiments [17,24], combined with the present study, support the use of ergocalciferol in the early stages of CKD not only to treat CKD associated bone mineral disorder but to improve vascular endothelial function and thus reduce CV risk in this patient group. The investigation of the pleotropic effects of ergocalciferol in uraemia requires further study to elucidate the optimum serum 25 (OH) D concentration to maximise endothelial function in patients with CKD and the same may be true in patients without evidence of kidney disease given that ergocalciferol improved vascular endothelial function in non-uraemic animals. This approach requires a shift in thinking away from the traditional use of ergocalciferol as a therapy to treat CKD BMD in towards further studies examining the mechanism and efficacy of vitamin D therapy on reducing cardiovascular risk in the early stages of CKD.

## Perspectives

- The present study was undertaken to evaluate the effect of ergocalciferol, a native form of vitamin D, on endothelial function in an *in vivo* model of mild uraemia – previous studies have used activated forms of vitamin D in more advanced uraemia or ESKD. Our study is unique in evaluating a low cost form of vitamin D with fewer side effects in earlier CKD where the vascular endothelium is more likely to be amenable to therapeutic intervention
- Ergocalciferol improves endothelial function and overcomes the detrimental effect of uraemia on the endothelium in a pre clinical model of mild CKD, independently of effects of blood pressure, calcium, phosphate or parathyroid hormone.
- This study supports the use of ergocalciferol in patients with mild to moderate chronic kidney disease as a therapeutic adjunct to improve endothelial function and thus reduce cardiovascular disease, which remains the major cause of death in such patients.

## Abbreviations

Ach: acetylcholine
CKD: chronic kidney disease
CV: cardiovascular
CVD: cardiovascular disease
ESKD: end stage kidney disease
HAEC: human aortic endothelial cells
PE: phenylephrine
PTH: parathyroid hormone
SNx: sub-total nephrectomy
SpNO: Spermin NONOate
VDRA: vitamin D receptor agonists
